# A core outcome set of patient-reported experience measures for primary care

**DOI:** 10.1186/s41687-026-01159-1

**Published:** 2026-08-06

**Authors:** Marie-Eve Poitras, Anne-Sophie Langlois, Marie-Eve Perron, Vanessa T. Vaillancourt, Maxime Sasseville

**Affiliations:** 1 Université de Sherbrooke, Sherbrooke, Canada; 2 Centre de recherche du CHUS, Centre Hospitalier Universitaire de Sherbrooke, Sherbrooke, Canada; 3 McGill University, Montreal, Canada; 4 Université Laval, Québec, Canada; 5 VITAM Research Center on Sustainable Health, Centre Intégré Universitaire de Santé et de Services Sociaux Capitale Nationale, Québec City, Canada

**Keywords:** Patient-reported experience measures, Primary care, Delphi study, Core outcome set, Care quality, Patient engagement, Communication, Accessibility, Coordination, Continuity, Comprehensiveness

## Abstract

**Background:**

Patient-Reported Experience Measures (PREMs) are validated self-reported perceptions of how patients experience care in a given context. Despite their recognized value, their integration in clinical settings is limited by conceptual heterogeneity and measurement inconsistency. To enhance comparability and operational clarity, this study aimed to develop a consensus-based core set of constructs representing the essential dimensions of patient experience in primary care.

**Methods:**

We conducted a three-round Delphi study in accordance with the COS-STAD and ACCORD methodological standards. We purposively recruited experts into three groups: patients, clinicians/decision-makers, and researchers. Sixteen constructs derived from a prior scoping review of validated PREMs were rated for inclusion in a core measurement set. Consensus thresholds were defined a priori as a positive median, interquartile range < 1, and < 5% negative ratings.

**Results:**

Eighty-nine experts participated across rounds (24 patients, 31 clinicians/decision-makers, 34 researchers). Of the sixteen constructs evaluated, six reached global consensus: *accessibility*,* continuity*,* coordination*,* communication*,* comprehensiveness*, and *patient engagement.* Subgroup analyses revealed convergence between patients and clinicians on these constructs in round 2, suggesting disciplinary heterogeneity in conceptualizing experience domains. Ten constructs did not achieve consensus, reflecting contextual or normative variability: including *equity*,* advocacy*,* organization*,* professional competencies*,* family context*, and *community context.*

**Conclusion:**

This Delphi-derived core set provides a consensus-informed basis for measuring patient experience in primary care. It offers a parsimonious, conceptually grounded framework to guide PREM development, support data comparability, and enhance responsiveness within learning health systems. Context-dependent constructs should complement the core set where local priorities or populations warrant expanded assessment.

**Supplementary Information:**

The online version contains supplementary material available at https://doi.org/10.1186/s41687-026-01159-1.

## Context and rationale

Patient-Reported Experience Measures (PREMs) are validated self-reported questionnaires that capture perceptions of how patients experience care in a given context [1–3]. The measures operationalize the quality of this perception in two domains: relationship with someone in the healthcare system (e.g., professional, administrative staff) and experience with organizational processes (e.g., access, safety of care, cleanliness) [1, 2, 4]. Patient experience is a fundamental dimension of care quality, adding to safety and effectiveness [5]. Because primary care relies on continuity, trust, and shared decision-making, assessing patient experience through validated data is essential to determine what influence engagement and outcomes [5]. Their integration in primary care clinical settings is constrained by the length and perceived complexity of existing instruments, which healthcare staff often regard as impractical for routine use [6, 7].

Their difficult pragmatic use is partly caused by substantial heterogeneity in both the conceptualization and measurement of patient-reported experience. A 2019 systematic review on the psychometric qualities of PREMs highlighted confusion between experience, satisfaction, and expectation constructs, describing PREMs as conceptually immature and inconsistent across contexts [1]. Recent concept analyses reveal an emerging convergence in defining attributes of patient experience, yet still highlight the strong influence of measurement context and overlapping constructs, which limit large-scale operationalization [5]. Developing a core set of PREMs could improve operationalization in primary care by achieving consensus on the essential constructs that should be measured to operationalize patient experience, strengthening the conceptual clarity and comparability in primary care research [8].

The Core Outcome Measures in Effectiveness Trials (COMET) initiative develops consensus-based core outcome sets (COS) that define the minimum outcomes to be measured and reported in specific contexts (www.comet-initiative.org) [9]. Designed initially to harmonize outcome reporting in clinical trials, the COS framework has since been extended to routine clinical care, audit, and health services research, where it supports consistency and comparability across studies and settings [10]. By identifying and reaching consensus on essential experience domains relevant to primary care, a core set can anchor measurement in a stable and operationally meaningful framework, enhancing measure choice, interpretability, comparability, and clinical utility while maintaining adaptability to local contexts [10].

In this study, PREMs refers to validated patient self-report instruments, domains to the operationalization of concepts those instruments target (e.g., accessibility, communication), and core outcome set to the minimum set of constructs that should be measured in a given context.

The goal of this study was to develop a consensus-based core set of patient-reported experience measures (PREMs) defining the essential domains for assessing patient experience in primary care.

## Methods

This study was planned in accordance with the Core Outcome Set–Standards for Development (*COS-STAD*) [11] and, because *COS-STAD* does not provide specific consensus method guidelines, the reporting of the Delphi process follows the *ACCORD* (ACcurate COnsensus Reporting Document) checklist for Conducting and Reporting of Delphi Studies [12]. The study is registered in the COMET Initiative database, study number 3096, (https://www.comet-initiative.org/Studies/Details/3096).

Aligned on the definition from *ACCORD*: “A Delphi study is a structured, iterative, multistage process used to gather and refine the judgments of a panel of experts through a series of questionnaires interspersed with controlled feedback.” [12]. The goal of a Delphi study is to attain collective agreement characterized by a convergence of opinions between a group of experts on a given subject, where no consensus had been established beforehand [13]. Our Delphi aimed to identify a core set of patient-reported experience measures and was guided by the *ACCORD* guideline. It included the following essential components: (1) structured, iterative, multiround process, (2) anonymous expert participation (3) controlled feedback, (4) quantitative aggregation and (5) predefined consensus criteria [12].

### Panel composition and recruitment

The panel of a Delphi study includes experts with in-depth knowledge or significant experience of the subject of interest [12, 13]. For this study, we recruited experts to be included in the panel if they were part of one of the following groups of interest: (a) a patient with lived experience or knowledge of primary care; (b) a person who acts as a primary care professional, decision-maker or coordinator; (c) a researcher with expertise in the field of primary care experience.

#### Sample size justification

In line with both the *ACCORD* reporting guidelines [12] and *COS-STAD* standards [11], the sample size for the Delphi panel was determined pragmatically to ensure diversity and balance across groups rather than a power analysis [13]. We aimed for a sample size of 75 experts (25 per group of interest) to capture a range of perspectives and to achieve response stability across rounds and this target was achieved cumulatively across all three rounds following the inclusive sampling method detailed in 2.1.2 [11, 13, 14].

#### Recruitment and attrition management

We used purposive and snowball sampling, to identify and invite experts to the panel [13]. First, we used the research team’s network to compile an initial list of 111 people to invite: including 15 patients, 45 coordinators, decision-makers and primary care professionals, and 51 researchers. We sent these potential experts an e-mail that described the purpose of the Delphi and informed them that they would shortly receive an electronic link to the first round Delphi questionnaire. We also invited them to share the invitation with their network to identify other additional potential experts (snowball sampling approach) [13]. We finally sent another e-mail including the questionnaire’s link to potential experts who expressed interest or did not reply to the first email.

As this strategy did not yield enough experts, we broadened our recruitment efforts. We contacted: 1) Patient partners groups, such as Canadian Primary Care Research Consortium Patient Council, to share our invitation through their social media platforms or newsletters to reach experts for group (a) a patient with lived experience or knowledge of primary care and; 2) Canadian and provincial primary care networks to invite them to share our recruitment to experts for group (b) primary care professional, decision-maker or coordinator. Finally, we conducted an exhaustive internet search to identify potential additional groups, associations or networks that could be interested in participating in this Delphi. This search led us to the identification of 68 additional potential experts.

Aligned with recent methodological research, we adopted an inclusive sampling approach for the Delphi rounds, which has been demonstrated to attain comparable consensus results [15]. This means that each response, even if isolated in a single Delphi round or incomplete, was considered valid. We invited all experts who met the eligibility criteria to take part to a round, regardless of their participation or not in previous rounds. This method enabled the broadest possible inclusion of experts within the processs and avoided the exclusion of minority or one-off opinions that could enrich the collective consensus. For experts joining in rounds 2 or 3 who had not participated previously, controlled feedback reflected the aggregated results of all prior respondents rather than their own previous answer.

### Consensus definition

Without specific consensus definition guidance, but in line with the *ACCORD* reporting guideline recommendations, the consensus in this Delphi study was defined as a priori and applied uniformly across all stakeholder groups of interest. An item was considered to have reached consensus for inclusion in the core set when (1) the central tendency was positive (2), the interquartile range (IQR) was less than 1 (3), fewer than 5% of experts provided a negative evaluation (from *somewhat disagree* to *strongly disagree*). In the reporting of this study, partial consensus refers to the consensus for inclusion for a specific group, but not for the entire sample.

### Questionnaire design, round structure and flow

We grounded this Delphi questionnaire through our prior scoping review of PREMs validated for primary care [16]. Our review identified 16 constructs linked to 23 PREMs: Accessibility, Communication, Coordination, Continuity, Comprehensiveness, Professional competencies, Equity, Advocacy, Organization, Family context, Team, Financial aspects, Relational aspects, Community context, Patient engagement, and Overall satisfaction. These constructs provided the empirical foundation for operational definitions and item generation in the first Delphi round (additional file 1).

We then conducted a face validity stage to adjust the operational definitions of the constructs included in Delphi [17]. Face validity assessment involves asking content experts to evaluate whether the operational definition of a construct adequately represents the concept it is intended to measure. To assess it, we administrated an online questionnaire presenting a proposed definition for each of the 16 identified constructs to three purposively selected experts. These three experts provided feedback on the proposed definitions and on the overall design of the questionnaire, as well as suggested adjustments through open-ended questions. The principal investigators (MEP, MS) independently reviewed the suggested adjustments and discussed any disagreements with the entire research team. All proposed modifications were subsequently discussed during a team meeting, and final decisions were made collectively (additional file 1).

Once the constructs and their definitions had been revised, we developed an online questionnaire using the REDCap platform [18]. We pre-tested the questionnaire with members of the extended team to identify and correct issues related to functionality (flow, branching logic), distribution, automated reminders, and typographical errors. At the beginning of the questionnaire, we included a complete description of the study and an electronic consent form. We used the piping option in REDCap to automate the individualized feedback in rounds two and three. In these rounds, experts were shown their previous answer alongside this heterogeneity may partly reflect the group’s current central tendency and were then asked to complete the ongoing round based on this information.

The Delphi was conducted over three consecutive rounds, each spaced approximately 30 days apart. The online questionnaire included closed-ended statements rated on a 7-point bipolar Likert scale, along with an optional free-text comment box for each item to allow experts to explain or contextualize their responses. For each construct, experts were asked to indicate the extent to which they believed the [construct] should be included in the measurement of patient experience in primary care (1 = strongly disagree to 7 = strongly agree).

In the second round, experts were asked to re-evaluate only those statements that had not reached consensus in the first round. Personalized feedback was provided to experts for each statement, showing how previous round responses compared with the aggregated results from all experts. The third round enabled experts to review the results of the second round, revise their responses if desired, and to further explain their level of agreement or disagreement to support their interpretation and contribute to the analysis of the Delphi results.

### Data analysis and feedback

For the qualitative analysis of comment from the face validity assessment, we used inductive thematic analysis [19]. A research professional (AL) conducted an open coding step on all the data from comments, then grouped codes into candidate themes [20].

A research professional (AL) used Microsoft Excel to manage data analysis tasks for central tendency and consensus attainment. For each item and subgroup (patients, primary care professional, decision-maker or coordinator, researchers), AL computed the median and interquartile range (IQR = Q3–Q1) with MEDIAN and QUARTILE.INC, and the percentages for each item. Consensus was defined a priori across all experts as: positive central tendency (median ≥ 5, IQR < 1, and < 5% negative ratings (1–3). Subgroup checks applied the same thresholds; items failing against any subgroup threshold were flagged for review. Missing ratings were handled by pairwise deletion per item. Free-text comments were summarized descriptively to inform wording changes; they were not used for scoring. All calculations were independently spot-checked (10% sample) by principal investigators (MS, MEP).

### Ethical considerations

We received an ethical certification from Université de Sherbrooke in August 2024, under reference 2024–4434. Prior to invitation, we submitted changes for the two extended recruitment strategies.

## Results

### Face validity assessment

Three experts participated in the face validity assessment of the Delphi questionnaire to examine the construct definitions and the overall questionnaire design. Three types (themes) of modifications emerged: (1) Revisions to the questionnaire structure, including splitting, reordering, transversal adjustments, and other structural refinements; (2) Modifications to constructs, including clarification or reorientation of content, addition of a dimension or substitution of elements; (3) Terminological revisions, both general and construct-specific. The complete modifications are presented in additional file 1.

### Experts panel description

A total of 89 experts took part in the Delphi (completed at least one round), and their characteristics are presented in Table 1. Of these, 24 (27%) belonged to group A (patients), 31 (35%) to group B (primary care professional, decision-maker or coordinator) and 34 (38%) to group C (researchers). Our panel of experts was predominantly represented by Canadian women between the ages of 35 and 44. Most of the experts (90%) did not identify as belonging to any equity-deserving group. Among those who did, 3% identified as First Nations, Inuit or Métis, 10% as members of racialized or visible minority communities, 16% as people with disabilities, and 8% as part of the LGBTQ2 + community; 4% preferred not to answer. Most had at least a university level degree. Experts’ attrition over the three rounds was 57 experts taking part in Round 1, 51 (10,6%) in Round 2 and 42 (26,4%) in Round 3. The panel was mainly composed of Canadian women between 35 and 44 years of age with a university-level education.

**Table 1:** Panel experts

Sociodemographic characteristics	*N* (%)
**Gender**	80 (90)
Female	64 (80)
Male	15 (19)
Prefer not to answer	1 (1)
**Age**	82 (92)
25 to 34	6 (7)
35 to 44	28 (34)
45 to 54	21 (26)
55 to 64	16 (20)
65 and over	11 (13)
**Education level**	83 (93)
No diploma	0 (0)
High school diploma	1(1)
College diploma	7 (8)
Bachelor’s degree	22 (27)
Master’s degree	14 (17)
Doctorate	35 (42)
Specialized graduate diploma	2 (2)
Other	2 (2)
**Membership of an equity group**	80 (90)
First Nations, Indigenous or Métis	2 (3)
Racialized communities or visible minorities	8 (10)
People with disabilities	13 (16)
LGBTQ2 + community	6 (8)
None	56 (70)
Prefer not to answer	3 (4)

### Round 1 consensus progress

The entire consensus attainment process is summarized in Table 2. In the first round of the Delphi, 57 experts took part, 8 of whom (14%) partially completed the questionnaire. Of the experts, 14 (25%) belonged to group A (patients), 16 (28%) to group B (primary care professional, decision-maker or coordinator), and 27 (47%) to group C (researchers). The panel had to rate 16 constructs. This first round of Delphi showed consensus attainment as subgroup analysis shows that group a (*n* = 14) reached consensus on accessibility, communication, coordination, continuity, comprehensiveness and overall satisfaction (6 constructs). Group B (*n* = 16) reached consensus on the patient engagement construct, and group C (*n* = 27) reached consensus on the continuity construct. None of the constructs was the subject of a consensus shared by all three groups (see additional file 2).

**Table 2:** Consensus attainment process

	Round 1 (*n* = 57)				Round 2 (*n* = 51)				Round 3 (*n* = 43)				Global consensus	
Accessibility	**Gr A**	**Gr B**	**Gr C**	**T (** ***n*** ** = 57)**	**Gr A**	**Gr B**	**Gr C**	**T (** ***n*** ** = 51)**	**Gr A**	**Gr B**	**Gr C**	**T**	**√**	
Median	7	7	7	7	7	7	7	7						
IQR	0,75	0,25	0,5	1	0	0	0	0						
Disagreement (%)	0	6,25	7,41	5,26	0	0	6,25	1,96						
Consensus	√				√	√		**√**						
Communication	**Gr A**	**Gr B**	**Gr C**	**T (** ***n*** ** = 55)**	**Gr A**	**Gr B**	**Gr C**	**T (** ***n*** ** = 50)**	**Gr A**	**Gr B**	**Gr C**	**T**	**√**	
Median	7	7	7	7	7	7	7	7						
IQR	0	1	1	1	0	0	0	0						
Disagreement (%)	0	7,14	7,41	5,45	0	0	12,50	4						
Consensus	√				√	√		**√**						
Coordination	**Gr A**	**Gr B**	**Gr C**	**T (** ***n*** ** = 55)**	**Gr A**	**Gr B**	**Gr C**	**T (** ***n*** ** = 50)**	**Gr A**	**Gr B**	**Gr C**	**T**	**√**	
Median	7	6,5	7	7	7	7	7	7						
IQR	0	1	1	1	0	0	0,25	0						
Disagreement (%)	0	7,14	3,70	3,64	0	0	6,25	2						
Consensus	√				√	√		**√**						
Continuity	**Gr A**	**Gr B**	**Gr C**	**T (** ***n*** ** = 54)**	**Gr A**	**Gr B**	**Gr C**	**T (** ***n*** ** = 50)**	**Gr A**	**Gr B**	**Gr C**	**T (** ***n*** ** = 43)**	**√**	
Median	7	6	7	7	7	7	7	7	7	7	7	7		
IQR	0	1	0,5	1	0	0	0	0	0	0	0,25	0		
Disagreement (%)	0	15,38	0	3,70	0	0	18,75	6	7,69	5,56	8,33	4,65		
Consensus	√		√		√	√						**√**		
Comprehensiveness	**Gr A**	**Gr B**	**Gr C**	**T (** ***n*** ** = 54)**	**Gr A**	**Gr B**	**Gr C**	**T (** ***n*** ** = 50)**	**Gr A**	**Gr B**	**Gr C**	**T**	**√**	
Median	7	6	7	7	7	7	7	7						
IQR	0,75	1	1	1	0	0	0	0						
Disagreement (%)	7,14	7,69	3,70	3,70	0	0	12,50	4						
Consensus					√	√		**√**						
Professional competencies	**Gr A**	**Gr B**	**Gr C**	**T (** ***n*** ** = 54)**	**Gr A**	**Gr B**	**Gr C**	**T (** ***n*** ** = 50)**	**Gr A**	**Gr B**	**Gr C**	**T (** ***n*** ** = 43)**	**X**	
Median	7	6	6	6	7	7	6	6,5	7	7	6,5	7		
IQR	0,75	1	2,5	2	1	1	2,25	1	0	1	3,25	1		
Disagreement (%)	7,14	7,69	18,52	12,96	0	0	18,75	6	7,69	5,56	25	11,63		
Consensus														
Equity	**Gr A**	**Gr B**	**Gr C**	**T (** ***n*** ** = 54)**	**Gr A**	**Gr B**	**Gr C**	**T (** ***n*** ** = 50)**	**Gr A**	**Gr B**	**Gr C**	**T (** ***n*** ** = 43)**	**X**	
Median	7	6	7	7	7	7	7	7	7	7	7	7		
IQR	0	1	0,5	1	0	0	0,5	0	0	0	1,75	1		
Disagreement (%)	7,14	7,69	7,41	7,41	0	0	12,50	6	7,69	5,56	25	11,63		
Consensus					√	√								
Advocacy	**Gr A**	**Gr B**	**Gr C**	**T (** ***n*** ** = 54)**	**Gr A**	**Gr B**	**Gr C**	**T (** ***n*** ** = 50)**	**Gr A**	**Gr B**	**Gr C**	**T (** ***n*** ** = 43)**	**X**	
Median	7	6	6	6	7	6	6	6	7	6	6	7		
IQR	1	0	3	2	1	0,75	3	1	0	1	1	1		
Disagreement (%)	0	15,38	18,52	12,96	0	5,56	31,25	10	7,69	5,56	16,67	9,30		
Consensus														
Organization	**Gr A**	**Gr B**	**Gr C**	**T (** ***n*** ** = 52)**	**Gr A**	**Gr B**	**Gr C**	**T (** ***n*** ** = 50)**	**Gr A**	**Gr B**	**Gr C**	**T (** ***n*** ** = 43)**	**X**	
Median	7	6,5	6	6	7	7	6	6	7	7	6	7		
IQR	1	1,25	2,5	1	1	1	1	1	0	1	2	1		
Disagreement (%)	0	0	18,52	9,62	0	0	18,75	6	7,69	16,67	16,67	13,95		
Consensus														
Family context	**Gr A**	**Gr B**	**Gr C**	**Tn = 51)**	**Gr A**	**Gr B**	**Gr C**	**T (** ***n*** ** = 50)**	**Gr A**	**Gr B**	**Gr C**	**T (** ***n*** ** = 43)**	**X**	
Median	7	6	6	6	7	7	6	7	7	7	6,5	7		
IQR	1	1,25	2	2	0	1	1,25	1	0	1	1,25	1		
Disagreement (%)	7,69	0	11,11	5,88	0	0	18,75	6	7,69	5,56	0	4,65		
Consensus														
Community context	**Gr A**	**Gr B**	**Gr C**	**Tn = 51)**	**Gr A**	**Gr B**	**Gr C**	**T (** ***n*** ** = 50)**	**Gr A**	**Gr B**	**Gr C**	**T (** ***n*** ** = 43)**	**X**	
Median	7	6	6	6	6,5	6	5,5	6	7	6	7	6,5		
IQR	1	2	2,5	2	1	1	1,25	1,75	1	1	1,25	1		
Disagreement (%)	7,69	8,33	18,52	13,73	0	0	6,25	4	7,69	5,56	0	4,65		
Consensus														
Relational aspects	**Gr A**	**Gr B**	**Gr C**	**T (** ***n*** ** = 50)**	**Gr A**	**Gr B**	**Gr C**	**T (** ***n*** ** = 50)**	**Gr A**	**Gr B**	**Gr C**	**T (** ***n*** ** = 43)**	**X**	
Median	7	7	7	7	7	7	7	7	7	7	7	7		
IQR	0	1	1	1	0,25	1	0,25	1	0	0,25	1	1		
Disagreement (%)	7,69	0	7,41	4	6,25	0	6,25	4	7,69	5,56	16,67	9,30		
Consensus														
Financial aspects	**Gr A**	**Gr B**	**Gr C**	**T (** ***n*** ** = 50)**	**Gr A**	**Gr B**	**Gr C**	**T (** ***n*** ** = 50)**	**Gr A**	**Gr B**	**Gr C**	**T (** ***n*** ** = 43)**	**X**	
Median	6	5.5	6	6	6	6	6	6	6	6	6	6		
IQR	2	3	1,5	2	0,25	1	1,25	1	0	1	1,25	1		
Disagreement (%)	15,38	16,67	18,52	18	0	11,10	18,75	10	0	5,56	16,67	6,98		
Consensus					√				√					
Team (Clinical and non-clinical)	**Gr A**	**Gr B**	**Gr C**	**T (** ***n*** ** = 50)**	**Gr A**	**Gr B**	**Gr C**	**T (** ***n*** ** = 50)**	**Gr A**	**Gr B**	**Gr C**	**T (** ***n*** ** = 42)**	**X**	
Median	7	6	6	6	6	6	6	6	7	6	6	6		
IQR	1	2	1,5	1	1	1	3	1	1	1	1	1		
Disagreement (%)	0	8,33	11,11	8	0	5,56	37,50	12	7,69	11,11	9,09	9,52		
Consensus														
Patient commitment	**Gr A**	**Gr B**	**Gr C**	**T (** ***n*** ** = 49)**	**Gr A**	**Gr B**	**Gr C**	**T (** ***n*** ** = 50)**	**Gr A**	**Gr B**	**Gr C**	**T**	**√**	
Median	7	7	7	7	7	7	7	7						
IQR	1	0	1,75	1	0	0	1	0						
Disagreement (%)	0	0	0	0	0	0	6,25	2						
Consensus		√			√	√		**√**						
Overall satisfaction	**Gr A**	**Gr B**	**Gr C**	**T (** ***n*** ** = 50)**	**Gr A**	**Gr B**	**Gr C**	**T (** ***n*** ** = 49)**	**Gr A**	**Gr B**	**Gr C**	**T (** ***n*** ** = 42)**	**X**	
Median	7	6	6	6	6	7	6	6	7	6,5	7	7		
IQR	0	1,25	1,5	2	1	1	1	1	1	1	1	1		
Disagreement (%)	0	8,33	11,11	8	0	5,56	0	2	0	5,56	0	2,38		
Consensus	√													

### Round 2 consensus progress

In this second round of the Delphi, 51 experts participated, of whom 2 (4%) partially completed the questionnaire. Of the experts, 16 (31%) belonged to group A (patients), 19 (37%) to group B (primary care professional, decision-maker or coordinator), and 16 to group C (researchers). Since none of the constructs presented in round 1 had achieved consensus, the number of constructs presented in round 2 remained the same. In total, 5 of the 16 constructs evaluated achieved global consensus at this round: *accessibility*,* communication*,* coordination*,* comprehensiveness*,* and patient engagement* (see Table 2). Group A reached partial consensus (in at least one group) on *equity*,* family context and financial aspects*, group B reached partial consensus on patient *engagement*,* accessibility*,* communication*,* coordination*,* continuity*,* comprehensiveness and equity*, while group C did not reach partial consensus on any constructs (Table 2).

### Round 3 consensus progress

In this third round of the Delphi, 43 experts participated, and one expert partially completed the questionnaire. Of these experts, 13 (30%) belonged to group A (patients), 18 (42%) to group B (clinicians, coordinator and decision-makers), and 12 (28%) to group C (researchers). Considering that consensus had been reached in round 2 for the constructs *accessibility*, *communication*, *coordination*, *comprehensiveness*, and *patient engagement*, a total of 11 constructs were presented in this round. An additional global consensus emerged for the *continuity* construct in Round 3. Group A also reached partial consensus on *financial aspects* and Groups b and c did not reach consensus on any of the 11 constructs evaluated (see Table 2).

## Discussion

Our goal was to bring experts together to develop a consensus-based core set of PREMs defining the essential domains for assessing patient experience in primary care. The three Delphi rounds quantified the iterative consensus attainment process of 89 experts with primary care expertise that included 24 patients (27%), 31 clinicians, coordinators or decision-makers (35%), and 34 researchers (38%). Of the 16 constructs initially presented, 6 achieved a global consensus (38%): *accessibility*,* continuity*,* communication*,* coordination*,* comprehensiveness and patient engagement.*

Experts profiles influenced intra-group consensus patterns. In Round 2, convergence between Groups A (patients) and B (clinicians, coordinators or decision-makers) was observed for *accessibility*, *communication*, *coordination*, *comprehensiveness*, and *patient engagement*, the same constructs that reached consensus at this stage. Group C (researchers) did not reach partial consensus on these constructs at round 3. This suggests that, by the second round, convergence had been achieved between patients and clinicians/decision-makers regarding core dimensions of care quality, accessibility, communication, coordination, comprehensiveness, and patient engagement, highlighting a shared prioritization of these constructs according to their perspective. Patient and clinician priorities do not always reliably align in multi-stakeholder Delphi studies [21]. With both groups converging in our study, it shows that these outcomes reflect a shared priority influenced by experiential knowledge. In contrast, the divergence of the researcher group suggests that they hold distinct perspectives on patient-experience-related constructs and greater internal heterogeneity of opinions. While all researchers were experts in primary care, this heterogeneity may partly reflect the diversity in their disciplinary backgrounds and theoretical viewpoints [22]. However, this may also reflect a misalignment between researchers’ perspectives, often encompassing a broad range of important but heterogeneous constructs, and those of clinicians or patients, whose viewpoints tend to be more grounded in concrete, day-to-day experiences of care delivery. Heterogeneous panels (such as those including patients, clinicians, and researchers) bring diverse epistemic perspectives and value systems that shape how constructs are interpreted and prioritized [23–25]. This diversity can enrich the consensus process and enhance the external validity of the results by ensuring that the final set of outcomes reflects multiple realities [23–25]. It has also been noted that greater diversity can reduce intra-group agreement and slow or limit the emergence of consensus, particularly when constructs relate to experience-based domains such as patient engagement or communication [25]. The aim of our Delphi was not to capture all possible viewpoints but to converge toward a core set of constructs that could serve as shared outcomes across multiple groups of interest. Reaching consensus on a limited number of constructs, but aligned with the viewpoints of the different groups of experts, was an intentional methodological goal aligned with recommendations for core set development, balancing inclusivity of perspectives with the need for operational clarity and applicability [11].

Other explanations for researcher heterogeneity cannot be excluded, including smaller subgroup sample sizes, differential attrition across rounds and the inclusive sampling strategy, which could have altered group composition between rounds.

The consensus attainment on *accessibility*,* continuity*,* coordination*,* communication comprehensiveness and patient engagement* constructs is aligned with consistent components of the classical definition of the measure of care quality and patient experience [26–29]. Four of the constructs evaluated in your Delphi (accessibility, continuity, coordination, and comprehensiveness) align directly with the core primary care evaluation identified by Starfield (commonly referred to as the 4Cs), integrated into the World Health Organization (WHO) Primary Health Care Measurement Framework [30, 31] and the Structural Indicators for the Strength of Primary Care (SiSPC) [32]. Donabedian’s classical framework of quality of care assessment also describes these constructs within the process dimension of quality, emphasizing their role in producing desirable patient outcomes through accessible, coordinated, and communicative care delivery with an engaged patient [33]. This identification as core components is also supported by the Institute of Medicine (IOM) identifying coordination, communication, and accessibility (via reduced delays and barriers) as essential for designing the patient–clinician relationship and organizing care delivery [34]. These findings also align with essential population level constructs, as described *in Systemic aspects of care delivery* domain of the SiSPC [32]. Foundational theories and recognized operationalization thus align with the “core” qualities of the results obtained in this consensus study.

In contrast, the expert panel identified *communication* and *patient engagement* (although not formally part of the Starfield 4Cs, the WHO or SiSPC) as “core” elements of patient experience measurement. Within the WHO measurement framework for primary care, it is people-centredness that is recognized as a key quality of care output (beside the 4Cs), emphasizing interpersonal processes such as trust, empathy, shared decision-making, and respect for patient preferences [31]. These processes partially overlap with *communication* and *patient engagement* conceptual definition [31]. Engagement interventions knowledge synthesis shows (education, shared decision-making, self-management support) improvements in knowledge, behaviour, and health status, grounding engagement as an enabling mechanism rather than a core primary-care quality measurement [35]. For communication, influences on outcome have been shown through established pathways (e.g., shared understanding, decision quality, therapeutic alliance, patient agency), which in turn support continuity and coordination, showing enablement qualities as well [36]. Concept analyses of continuity of care also position communication as an enabling mechanism of continuity and coordination [37]. Engagement and communication are cross-cutting mechanisms that produce care quality (4Cs) during delivery, while the 4Cs define primary care functions. While patient experience is part of overall care quality assessment, it extends beyond it by capturing core mechanisms of patient experience, communication and engagement, enabling accessible, coordinated and comprehensive primary care. Their inclusion by the expert panel reflects the evolution of primary care models from functional components to a relational, person-centred paradigm that integrates both process and experiential measurement of care quality [38–40].

Evidence from knowledge syntheses shows that primary care experience on consensual constructs affects outcomes. Continuity is associated with lower mortality across multiple observational studies in a systematic review [41]. Communication quality improves adherence and patient-centred outcomes in meta-analyses and rapid reviews [42, 43]. Coordination interventions show benefits for patient experience and, in some contexts, reduced services utilization in systematic reviews [44, 45]. Accessibility improvements, including first-contact access and scheduling changes, are linked to lower ambulatory care-sensitive hospitalizations and shorter waits in a systematic review [46]. Recent syntheses also link access, continuity, comprehensiveness and coordination (as part of integrated care) to stronger primary care functions to better health outcomes and lower costs [47]. These syntheses also suggest that the six “core” constructs identified by our Delphi panel likely serve key operational roles, functioning as proximal mechanisms through which measured constructs translate into distal impacts on care quality and outcomes.

The ten constructs that did not reach global consensus (professional competencies, equity, advocacy, organization, family context, team, financial aspects, relational aspects, community context, and overall satisfaction) represent domains that are either context-dependent or interpretively diffuse, which often limits convergence in Delphi processes [23–25]. Constructs such as *family context*, *community context*, and *financial aspects* vary widely across health systems, regions, and patient groups. Their perceived importance depends on contextual realities (e.g., insurance models, social care integration, family involvement norms). Similar patterns of divergence have been observed in Delphi studies of health service quality where contextual constructs failed to reach consensus due to location or institutional variability [23, 24]. Constructs like *relational aspects*, *professional competencies*, and *team* can overlap with broader constructs like *communication* and *coordination*. For constructs that share definition boundaries, experts could have interpreted them differently (e.g., separate domains, embedded), making global consensus difficult. Constructs with strong normative, governance, or value implications (e.g., equity, advocacy, organization) are more likely to elicit divergent interpretations and polarized responses in expert panels. A study on public involvement in health research showed that key groups bring normative expectations, ethical frameworks, and institutional perspectives to how these constructs should be defined and operationalized, making global consensus difficult [48].

### Clinical implications

The six constructs that reached consensus (accessibility, continuity, coordination, communication, comprehensiveness, and patient engagement) provide a consensus-informed basis for future PREM development, selection and use in primary care. Their alignment with foundational frameworks (4Cs, WHO Primary Health Care Measurement Framework, SiSPC, PaRIS OECD framework [49]) strengthens the conceptual grounding of this shortlist and suggests its potential utility for guiding local quality improvement and system-level monitoring, pending psychometric validation. Future work could integrate validated instruments built around these domains into routine clinical workflows, allowing health teams to collect brief, meaningful experience data [50]. In addition to patient-reported assessment, each construct can be linked to primary care components: accessibility to appointment systems and first-contact services; continuity to relational and informational consistency; coordination to care integration and referrals; communication and engagement to shared decision-making and trust; and comprehensiveness to the breadth of services provided. Future work could embed validated instruments built around these domains into primary care dashboards and patient portals to support continuous feedback loops within learning health systems, with potential to inform practice adaptation, training priorities, and equity monitoring. Patient’s involvement in this study and in the consensus of core constructs to be included in the patient experience measurement makes the measure more relevant to clinicians. Clinicians have been shown to be more inclined to include self-reported measurement if patients recognized the relevance of the constructs used [51].

### Implications for research

The core set of this study will also guide researchers in the development of standardized, evidence-based tools to reliably measure patient experience in primary care. There is a need to refine and validate this core set across diverse primary care settings, leading to a psychometric evaluation (e.g., reliability, validity, sensitivity to change, measurement invariance). Comparative evaluations could determine how the core constructs perform across delivery models, health systems, and cultural contexts. Longitudinal research could explore how PREMs evolve as care delivery shifts toward digital, team-based, and community-integrated models, ensuring that patient-experience measurement remains relevant and adaptive. It is also important to consider constructs that did not reach global consensus, which were all identified in existing measurements in the scoping review and remain conceptually relevant for the assessment of patient experience [16]. While these constructs elicited more variability across key groups, they influence how patients experience care and can capture contextually relevant determinants not reflected in the core set. Their inclusion could therefore be justified in specific evaluative or research settings where these dimensions are particularly relevant. Combined with the fundamental constructs identified through consensus, these context-dependent domains can enrich data collection and interpretation when integrated selectively by clinical or research teams.

### Strengths and limitations

The Delphi process followed recognized methodological standards (*COS-STAD* and *ACCORD*), applied predefined consensus thresholds, and involved a diverse, balanced panel representing patients, clinicians, coordinator, decision-makers, and researchers. The iterative feedback process and subgroup analyses allowed for transparent, reproducible tracking of consensus attainment. We attained the a priori sample size and retained an acceptable level of participation with less than 30% overall attrition.

Limitations must be acknowledged. Applying the COS framework to patient experience instead of classical biological or clinical outcomes can be seen as an overextension of the methodology. However, several domains evaluated in this project (among others, but not exclusively, accessibility, continuity, coordination, comprehensiveness) are also classical primary care quality dimensions and thus this study addresses them as patient-reported assessments of those processes rather than as structural or process indicators of system performance. Non-probabilistic sampling and the predominance of experts from a single national context could limit generalizability. This limit is mitigated by the alignment of the results on conceptual and operationalized definitions of care quality, improving the external validity of the panel consensus. Despite using an inclusive sampling analytic and approach, attrition between rounds could have influenced stability estimates. The panel was mainly composed of educated, Canadian women between 35 and 44 years of age and it may limit the representativeness of the consensus for populations with lower literacy, limited digital access, or from equity-deserving groups. The face-validity assessment relied on three purposively selected experts, which constrains the thoroughness of the construct-definition refinement step. The face-validity assessment relied on only three purposively selected experts, which constrains the thoroughness of the construct-definition refinement step. Our study relied on pooled consensus attainment. When subgroup consensus was not achieved, pooled agreement may have masked disagreements among stakeholder groups. Although inclusive sampling has previously been shown not to affect consensus attainment while improving representation, these findings have not been replicated, and we could have reached a different consensus without inclusive sampling [52]. Online data collection and high general literacy levels from definitions may have reduced participation from populations with lower general and digital literacy. Some constructs presented conceptual overlap, which future studies should disentangle through field-testing and cognitive interviewing. Finally, while consensus was achieved on core constructs, validation in real-world primary care settings is needed to confirm their predictive and practical utility.

## Conclusion

This Delphi study identified six core outcome domains (accessibility, continuity, coordination, communication, comprehensiveness, and patient engagement) representing what should be operationalized for measuring patient experience in primary care. These constructs are consistent with foundational frameworks of primary care quality and collectively provide a consensus-informed basis for operationalizing patient experience across clinical and research contexts. Further field testing and psychometric validation are required before they can be adopted as a standardized framework. By distinguishing between constructs related to care delivery quality as well as relational mechanisms, the study clarifies the scope and structure of patient experience measurement in primary care.

While some constructs did not reach global consensus, their contextual relevance remains important. Constructs such as equity, advocacy, organization, and relational or community context capture system-level and preference-based determinants of patient experience and should be selectively included when aligning with local or population priorities. This adaptable approach can offer a practical and scalable framework for improving comparability while maintaining flexibility across diverse care settings.

This consensus-informed core provides a basis for harmonized measurement, evidence synthesis, and feedback integration in learning health systems. Its use can advance research and practice by fostering more coherent data collection, enabling cross-study comparability, and supporting responsive, people-centred primary care design.

## Supplementary Information

Below is the link to the electronic supplementary material.

Supplementary Material 1

Supplementary Material 2

## Data Availability

Raw data for the entire project can be obtained by contacting the corresponding author.
