# Supplementary material for: A core outcome set of patient-reported experience measures for primary care
Source: J Patient Rep Outcomes. 2026 Aug 6;10:205. doi: 10.1186/s41687-026-01159-1 (PMC13745604; doi:10.1186/s41687-026-01159-1)
Supplement: Supplementary file 1 [file 41687_2026_1159_MOESM1_ESM.docx]

## Annexe 1 – Apparent validity round – Review of constructs and their definitions

| **Constructs**  **commented** | **Initial definitions** | **Comments that led to a revision** | **Definitions presented** |
| --- | --- | --- | --- |
| Accessibility | L’accessibilité, en tant que construit, est définie par la combinaison des deux définitions suivantes :  Premier contact : La facilité avec laquelle une personne peut obtenir les soins nécessaires (y compris des conseils et du soutien) auprès de la clinicienne ou du clinicien de son choix dans un délai approprié à l’urgence du problème.  Accessibilité organisationnelle : La façon dont les ressources en soins de santé primaires sont organisées pour assurer l’accessibilité à une grande diversité de patientes et patients à entrer en contact avec les cliniciennes et cliniciens et à accéder aux services de santé. | « Le terme premier contact porte à confusion – Devrions davantage évoquer suivi/accompagnement »  « Préciser la notion de localisation. » | L’accessibilité, en tant que construit, est définie par la combinaison des deux définitions suivantes :  **Contact** : La facilité avec laquelle une personne peut obtenir les soins nécessaires (y compris des conseils et du soutien) auprès de la clinicienne ou du clinicien de son choix dans un délai approprié à l’urgence du problème.  Accessibilité organisationnelle : La façon dont les ressources en soins de santé primaires sont organisées, **incluant leur facilité d'accès géographique**, pour assurer l’accessibilité à une grande diversité de patientes et patients à entrer en contact avec les cliniciennes et cliniciens et à accéder aux services de santé. |
| Communication | Communication interpersonnelle : La capacité de la clinicienne ou du clinicien à bien identifier et comprendre les préoccupations de la patiente ou du patient, à expliquer les enjeux liés aux soins de santé et à s'engager dans une prise de décision partagée, si c’est ce qui est souhaité. | - | Communication interpersonnelle : La capacité de la clinicienne ou du clinicien à bien identifier et comprendre les préoccupations de la patiente ou du patient, à expliquer les enjeux liés aux soins de santé et à s'engager dans une prise de décision partagée, si c’est ce qui est souhaité. |
| Coordination | Continuité de gestion : La prestation de services par différentes et/ou différents cliniciens et/ou cliniciennes en temps voulus et de manière complémentaire, assurant ainsi des soins connectés et cohérents. | « Soins connectés ne me dit rien. Soins prodigués en continu et cohérents… pas sûre. » | Continuité de gestion : La prestation de services par différentes et/ou différents cliniciens et/ou cliniciennes en temps voulus et de manière complémentaire, assurant ainsi des soins **interreliés** et cohérents. |
| Continuity | La continuité, en tant que construit, est définie par la combinaison des deux définitions suivantes :  Informationnelle : Le degré auquel les informations sur les soins passés sont utilisées pour rendre les soins actuels adaptés à la patiente ou au patient.  Relationnelle : La relation thérapeutique entre une patiente ou un patient et un ou plusieurs cliniciennes ou cliniciens qui couvre divers événements de soins de santé et aboutit à une meilleure connaissance de la patiente ou du patient, permettant des soins cohérents avec ses besoins. | « Définition informationnelle difficile à comprendre : Le degré auquel les informations sur les soins passés sont utilisées pour rendre les soins actuels adaptés à la patiente ou au patient. » | La continuité, en tant que construit, est définie par la combinaison des deux définitions suivantes :  Informationnelle : Le degré auquel les informations sur les soins **obtenus** **dans le passé** sont utilisées pour **adapter** les soins actuels **aux besoins spécifiques de la patiente ou du patient**.  Relationnelle : La relation thérapeutique entre une patiente ou un patient et un ou plusieurs cliniciennes ou cliniciens qui couvre divers événements de soins de santé et aboutit à une meilleure connaissance de la patiente ou du patient, permettant des soins cohérents avec ses besoins. |
| Comprehensiveness | Intégralité, en tant que construit, est définie par la combinaison des deux définitions suivantes :  Exhaustivité des services : La prestation, directe ou indirecte, d'une gamme complète de services pour répondre aux besoins en soins de santé des patientes et des patients. Cela inclut la promotion de la santé, la prévention, le diagnostic et le traitement des affections courantes, l'orientation vers d'autres cliniciennes et cliniciens, la gestion des maladies chroniques, la réadaptation, les soins palliatifs et, dans certains modèles, les services sociaux.  Soins centrés sur la personne dans sa globalité : Le degré auquel une clinicienne ou un clinicien identifie correctement et prend en compte les aspects physiques, émotionnels et sociaux de la santé d’une patiente ou d’un patient, tout en considérant le contexte communautaire dans la prestation des soins à la patiente ou au patient. | « Nous devirons peut-être utiliser le terme '' Intégralité des services '' plutôt que ''Exhaustivité des services'' ? Voir l.24 » | Intégralité, en tant que construit, est définie par la combinaison des deux définitions suivantes :  **Intégralité** des services : La prestation, directe ou indirecte, d'une gamme complète de services pour répondre aux besoins en soins de santé des patientes et des patients. Cela inclut la promotion de la santé, la prévention, le diagnostic et le traitement des affections courantes, l'orientation vers d'autres cliniciennes et cliniciens, la gestion des maladies chroniques, la réadaptation, les soins palliatifs et, dans certains modèles, les services sociaux.  Soins centrés sur la personne dans sa globalité : Le degré auquel une clinicienne ou un clinicien identifie correctement et prend en compte les aspects physiques, émotionnels et sociaux de la santé d’une patiente ou d’un patient, tout en considérant le contexte communautaire dans la prestation des soins à la patiente ou au patient. |
| Professional skills | Compétences professionnelles - Qualité technique des soins cliniques : Le degré auquel les procédures cliniques reflètent les évidences scientifiques actuelles et/ou respectent les normes établies pour le contenu technique ou les compétences. | « Ici je pense qu'il faille se référer à des auteurs pour appuyer notre définition. Que disent les auteurs de type Gold Standard sur la définition de compétence professionnelle ? Ainsi je ne suis pas à l'aise de modifier la définition sans nous appuyer. » | **Qualité des soins cliniques** : Le degré auquel les procédures cliniques reflètent les évidences scientifiques actuelles et/ou respectent les normes établies pour le contenu technique ou les compétences **relationnelles**. |
| Equity | Équité - Le degré auquel l'accès aux soins de santé et à des services de qualité sont fournis en fonction des besoins de santé, sans différences systémiques basées sur des caractéristiques individuelles ou sociales. | « Songer à ajouter ''et en tenant compte des particularités/réalités'' » | Équité - Le degré auquel l'accès aux soins de santé et à des services de qualité sont fournis en fonction des besoins de santé, sans différences systémiques basées sur des caractéristiques individuelles ou sociales **et en tenant compte de l'identité et de la réalité de chaque personne.** |
| Organization | Organisation, en tant que construit, est définie par la combinaison des quatre définitions suivantes :  Intégration des systèmes : Le degré auquel l’organisation de l’établissement de soins de santé a établi et maintien des liens avec d’autres parties du système de soins de santé et des services sociaux pour faciliter le transfert des soins et coordonner les soins concomitants entre différentes organisations de soins de santé.  Processus d'amélioration de la qualité : L'institutionnalisation de politiques et de procédures qui fournissent une rétroaction sur les structures et les pratiques permettant l’amélioration de la qualité clinique des soins et assurant la sécurité.  Gestion de l'information clinique : L’adéquation des méthodes et des systèmes permettant de capturer, mettre à jour, récupérer et surveiller les données des patientes et des patients en temps opportuns et de manière pertinente et confidentielle.  Environnement physique : Il se rapporte aux espaces physiques où les services de santé sont fournis. Notamment, l'espace, le confort, la propreté, la clarté des affiches informationnelles pour faciliter l'orientation des patientes et patients dans les établissements de soins de santé, etc., sont considérés comme des sources importantes de satisfaction. | « Soins concomitants (simultané ou synchrones) insti, institutionnalisation (implantation) adéquation (cohérence) sont des termes bien savants. Je vous en propose d'autres plus clairs selon moi entre le (). » | Organisation, en tant que construit, est définie par la combinaison des quatre définitions suivantes :  Intégration des systèmes : Le degré auquel l’organisation de l’établissement de soins de santé a établi et maintien des liens avec d’autres parties du système de soins de santé et des services sociaux pour faciliter le transfert des soins et coordonner les soins **simultanés** entre différentes organisations de soins de santé.  Processus d'amélioration de la qualité : L**'implantation** de politiques et de procédures qui fournissent une rétroaction sur les structures et les pratiques permettant l’amélioration de la qualité clinique des soins et assurant la sécurité.  Gestion de l'information clinique : La **cohérence** des méthodes et des systèmes permettant de capturer, mettre à jour, récupérer et surveiller les données des patientes et des patients en temps opportuns et de manière pertinente et confidentielle.  Environnement physique : Il se rapporte aux espaces physiques où les services de santé sont fournis. Notamment, l'espace, le confort, la propreté, la clarté des affiches informationnelles pour faciliter l'orientation des patientes et patients dans les établissements de soins de santé, etc., sont considérés comme des sources importantes de satisfaction. |
| Advocacy | Défense des droits - Le degré auquel les cliniciennes et cliniciens défendent les meilleurs intérêts des patientes et des patients individuels et des groupes de patientes et patients en matière de santé (y compris les déterminants sociaux de la santé) et de soins de santé. | « Pas sûre que c'est clair entre défense des droits et militance ou advocacy je vois une différence. On peut se battre pour un des patients (défendre le droit à un service par exemple) ou encore prendre position publiquement (militer). Je préciserais ce qu'est votre définition de la défense des droits. » | Défense des droits - Le degré auquel les cliniciennes et cliniciens défendent les meilleurs intérêts des patientes et des patients individuels et des groupes de patientes et patients en matière de santé (y compris les déterminants sociaux de la santé) et de soins de santé. **Cette défense des intérêts ne nécessite pas nécessairement une prise de position militante ou publique, mais se traduit par un engagement professionnel et éthique à promouvoir le bien-être des patientes et patients.** |
| Family context | Contexte familial - Soins centrés sur la famille : Le degré auquel la clinicienne ou le clinicien prend en compte la famille (sous toutes ses formes) et comprend son influence sur la santé d'une personne, en l'engageant comme partenaire dans les soins de santé continus. | « Le terme ''famille'' pourrait être un peu réducteur, songer à un terme comme ''support system'', proches, etc. » | Soins centrés sur la famille **et les proches** : Le degré auquel la clinicienne ou le clinicien prend en compte la famille (sous toute ses formes) **et les proches**, et comprend leur influence sur la santé d'une personne, en l'engageant comme partenaire dans les soins de santé continus. |
| Community context | Contexte communautaire, en tant que construit, est définie par la combinaison des deux définitions suivantes :  Participation des patientes et patients de la communauté : L'implication des clientes et clients et des membres de la communauté dans les décisions concernant la structure de la clinique et des services fournis (par exemple, les comités consultatifs, la gouvernance communautaire)  Considération du contexte populationnel : Le degré auquel les cliniciennes et cliniciens en soins primaires évaluent et répondent aux besoins de santé de la population qu'ils desservent. | « Il est important d'aller au-delà de la participation/consultation et de privilégier l'approche de partenariat qui est basée sur la complémentarité des savoirs professionnels et des personnes expertes de vécu (Ex. vie avec la maladie, etc.) »  « Je changerais le terme client pour citoyen. Le contexte populationnel est en lien direct avec la connaissance du milieu desservi par les professionnels ainsi que la réalité socio-démographique du milieu. » | Contexte communautaire, en tant que construit, est définie par la combinaison des deux définitions suivantes :  Participation des patientes et patients de la communauté : **Implication de la communauté dans les décisions sur la structure de la clinique et les services fournis, selon une approche partenariale valorisant la complémentarité des savoirs professionnels et expérientiels.**  Considération du contexte populationnel : Le degré auquel les cliniciennes et cliniciens en soins primaires évaluent et répondent aux besoins de santé de la population qu'ils desservent. |
| Relational aspects | Aspects relationnels, en tant que construit, est définie par la combinaison des deux définitions suivantes :  Respect : Le degré auquel les professionnels et professionnelles de la santé et le personnel de soutien répondent aux attentes des utilisatrices et utilisateurs en matière de traitement interpersonnel, font preuve de respect envers la dignité des patientes et des patients et garantissent une confidentialité adéquate.  Confiance : Le niveau de confiance que les patientes et patients accordent aux professionnels et professionnelles de la santé. | « Difficile de comprendre le terme ''traitement interpersonnel'' » | Aspects relationnels, en tant que construit, est définie par la combinaison des deux définitions suivantes :  Respect : Le degré auquel les professionnels et professionnelles de la santé et le personnel de soutien répondent aux attentes des utilisatrices et utilisateurs en matière de **relations humaines**, font preuve de respect envers la dignité des patientes et des patients et garantissent une confidentialité adéquate.  Confiance : Le niveau de confiance que les patientes et patients accordent aux professionnels et professionnelles de la santé. |
| Financial aspects | Aspects financiers, en tant que construit, est définie par la combinaison des deux définitions suivantes :  Efficacité/productivité : Atteindre les résultats souhaités avec l'utilisation des ressources la plus rentable en termes de coût-efficacité.  Accès financier : Évaluation du montant que la patiente ou le patient paie pour les consultations médicales et le traitement prescrit. | - | Aspects financiers, en tant que construit, est définie par la combinaison des deux définitions suivantes :  Efficacité/productivité : Atteindre les résultats souhaités avec l'utilisation des ressources la plus rentable en termes de coût-efficacité.  Accès financier : Évaluation du montant que la patiente ou le patient paie pour les consultations médicales et le traitement prescrit. |
| Team | Équipe (clinique et non-clinique), en tant que construit, est définie par la combinaison des deux définitions suivantes :  Équipe intersectorielle : Le degré auquel la clinicienne ou le clinicien en soins primaires collabore avec des praticiennes et praticiens d'autres secteurs que la santé pour fournir des services ayant un impact sur la santé.  Équipe multidisciplinaire : Des praticiennes et praticiens de diverses disciplines de santé collaborent pour fournir des soins continus. | « Ajouter la reconnaissance du patient comme expert de la vie avec la maladie et donc comme membre de l'équipe (Ex. multidisciplinaire) » | Équipe (clinique et non-clinique), en tant que construit, est définie par la combinaison des deux définitions suivantes :  Équipe intersectorielle : Le degré auquel la clinicienne ou le clinicien en soins primaires collabore avec des praticiennes et praticiens d'autres secteurs que la santé pour fournir des services ayant un impact sur la santé.  Équipe multidisciplinaire : Des praticiennes et praticiens de diverses disciplines de santé **collaborent avec la patiente ou le patient, considéré comme un partenaire actif dans ses soins** pour offrir des soins continus et adaptés. |
| Patient engagement | Engagement patient - Le désir et la capacité de choisir de participer activement aux soins de manière spécifiquement adaptée à chaque individu, en coopération avec une ou un prestataire de soins ou une institution, dans le but de maximiser les résultats ou d'améliorer l'expérience des soins. | « Remplacer ''coopération'' par ''partenariat'' »  « Ajouter une dimension de reconnaissance des savoirs et de la pratique du patient/client dans ses propres soins. » | **L’engagement patient se définit comme étant un processus de transformation individuelle ou organisationnelle qui permet au patient ou à la patiente et ses proches aidants d'entrer en relation avec différents acteurs de la santé. (DCPP et CIO-UDEM, 2016) L’engagement patient correspond au désir et et à la capacité de la personne à choisir activement son niveau de participation aux soins d'une manière qui lui est propre, en coopération avec un prestataire ou une institution de soins de santé, dans le but de maximiser ses résultats ou d’améliorer ses expériences de soins. (Higgins T, Larson E, et Schnall R, 2017)** |
| Overall satisfaction | Satisfaction globale - Sentiment de sécurité et d’avoir un suivi adéquat, ainsi que satisfaction à l'égard du soutien émotionnel, des informations de santé, du contrôle de la prise de décision et des compétences des professionnelles et professionnels de la santé. |  | Satisfaction globale - Sentiment de sécurité et d’avoir un suivi adéquat, ainsi que satisfaction à l'égard du soutien émotionnel, des informations de santé, du contrôle de la prise de décision et des compétences des professionnelles et professionnels de la santé. |
| General comments |  | « L'utilisation du terme ''construit'' est peu compréhensible pour Mrs et Mme tout le monde. »  « Modification de la phrase suivante : Le construit X prend forme grâce à l'association des X définitions suivantes »  « Modification de certaines questions : à quel point êtes-vous en accord avec le fait d'intégrer le construit X dans la mesure de l'expérience des patientes et patients en soins primaires ? »  « Traduire Comprehensiveness par Intégralité plutôt que par exhaustivité. » | Ajout d’une définition au terme construit en début de questionnaire.  [Construit ex L'accessibilité], en tant que construit, est définie par la combinaison des [Nbr] définitions suivantes :  À quel point êtes-vous en accord avec le fait d'intégrer X (ex l'accessibilité) dans la mesure de l'expérience des patientes et patients en soins primaires ?  **Intégralité**, en tant que construit, est définie par la combinaison deux définitions suivantes :  **Intégralité** des services : La prestation, directe ou indirecte, d'une gamme complète de services pour répondre aux besoins en soins de santé des patientes et des patients. Cela inclut la promotion de la santé, la prévention, le diagnostic et le traitement des affections courantes, l'orientation vers d'autres cliniciennes et cliniciens, la gestion des maladies chroniques, la réadaptation, les soins palliatifs et, dans certains modèles, les services sociaux. |
