# Supplementary material for: A core outcome set of patient-reported experience measures for primary care
Source: J Patient Rep Outcomes. 2026 Aug 6;10:205. doi: 10.1186/s41687-026-01159-1 (PMC13745604; doi:10.1186/s41687-026-01159-1)
Supplement: Supplementary file 2 [file 41687_2026_1159_MOESM2_ESM.docx]

*Translation notice: This is an English translation of the original French document, prepared for publication purposes only. The translation has not been formally validated. In the event of any discrepancy, the French original takes precedence.*

**Additional File 1 – Face-validity round: review of constructs and definitions**

| **Construct** | **Initial definition** | **Comments that led to revision** | **Definition presented** |
| --- | --- | --- | --- |
| **Accessibility** | Accessibility, as a construct, is defined by the combination of the following two definitions:  First contact: The ease with which a person can obtain necessary care (including advice and support) from the clinician of their choice within a timeframe appropriate to the urgency of the problem.  Organizational accessibility: The way in which primary healthcare resources are organized to ensure accessibility for a wide diversity of patients to contact clinicians and access health services. | *"The term first contact is confusing – We should refer more to follow-up/accompaniment."*  *"Specify the notion of location."* | Accessibility, as a construct, is defined by the combination of the following two definitions:  Contact: The ease with which a person can obtain necessary care (including advice and support) from the clinician of their choice within a timeframe appropriate to the urgency of the problem.  Organizational accessibility: The way in which primary healthcare resources are organized, including ease of geographic access, to ensure accessibility for a wide diversity of patients to contact clinicians and access health services. |
| **Communication** | Interpersonal communication: The ability of the clinician to correctly identify and understand the patient's concerns, explain health care issues, and engage in shared decision-making, if desired. | *–* | Interpersonal communication: The ability of the clinician to correctly identify and understand the patient's concerns, explain health care issues, and engage in shared decision-making, if desired. |
| **Coordination** | Management continuity: The provision of services by different clinicians in a timely and complementary manner, thus ensuring connected and coherent care. | *"Connected care does not mean anything to me. Care provided continuously and coherently… not sure."* | Management continuity: The provision of services by different clinicians in a timely and complementary manner, thus ensuring interrelated and coherent care. |
| **Continuity** | Continuity, as a construct, is defined by the combination of the following two definitions:  Informational: The degree to which information about past care is used to make current care adapted to the patient.  Relational: The therapeutic relationship between a patient and one or more clinicians that covers various health care events and results in better knowledge of the patient, enabling care consistent with their needs. | *"Informational definition difficult to understand: The degree to which information about past care is used to make current care adapted to the patient."* | Continuity, as a construct, is defined by the combination of the following two definitions:  Informational: The degree to which information about care received in the past is used to adapt current care to the specific needs of the patient.  Relational: The therapeutic relationship between a patient and one or more clinicians that covers various health care events and results in better knowledge of the patient, enabling care consistent with their needs. |
| **Comprehensiveness** | Comprehensiveness, as a construct, is defined by the combination of the following two definitions:  Comprehensiveness of services: The direct or indirect provision of a full range of services to meet the healthcare needs of patients. This includes health promotion, prevention, diagnosis and treatment of common conditions, referral to other clinicians, chronic disease management, rehabilitation, palliative care and, in some models, social services.  Whole-person-centred care: The degree to which a clinician correctly identifies and takes into account the physical, emotional and social aspects of a patient's health, while considering the community context in the provision of care to the patient. | *"We might want to use the term 'Comprehensiveness of services' rather than 'Exhaustiveness of services'? See line 24."* | Comprehensiveness, as a construct, is defined by the combination of the following two definitions:  Comprehensiveness of services: The direct or indirect provision of a full range of services to meet the healthcare needs of patients. This includes health promotion, prevention, diagnosis and treatment of common conditions, referral to other clinicians, chronic disease management, rehabilitation, palliative care and, in some models, social services.  Whole-person-centred care: The degree to which a clinician correctly identifies and takes into account the physical, emotional and social aspects of a patient's health, while considering the community context in the provision of care to the patient. |
| **Professional skills** | Professional skills – Quality of technical clinical care: The degree to which clinical procedures reflect current scientific evidence and/or meet established standards for technical content or skills. | *"Here I think we need to refer to authors to support our definition. What do Gold Standard authors say about the definition of professional competence? I am not comfortable modifying the definition without support."* | Quality of clinical care: The degree to which clinical procedures reflect current scientific evidence and/or meet established standards for technical content or relational skills. |
| **Equity** | Equity – The degree to which access to healthcare and quality services are provided according to health needs, without systemic differences based on individual or social characteristics. | *"Consider adding 'and taking into account particularities/realities'."* | Equity – The degree to which access to healthcare and quality services are provided according to health needs, without systemic differences based on individual or social characteristics, and taking into account each person's identity and reality. |
| **Organization** | Organization, as a construct, is defined by the combination of the following four definitions:  Systems integration: The degree to which the healthcare organization has established and maintained links with other parts of the healthcare system and social services to facilitate care transfer and coordinate concurrent care between different healthcare organizations.  Quality improvement process: The institutionalization of policies and procedures that provide feedback on structures and practices enabling the improvement of the clinical quality of care and ensuring safety.  Clinical information management: The adequacy of the methods and systems to capture, update, retrieve and monitor patient data in a timely, relevant and confidential manner.  Physical environment: Refers to the physical spaces where health services are provided. Notably, space, comfort, cleanliness, and clarity of informational signage to facilitate patient orientation in healthcare facilities are considered important sources of satisfaction. | *"Concurrent care (simultaneous or synchronous), institutionalization (implementation), adequacy (coherence) are quite technical terms. I propose clearer alternatives in parentheses."* | Organization, as a construct, is defined by the combination of the following four definitions:  Systems integration: The degree to which the healthcare organization has established and maintained links with other parts of the healthcare system and social services to facilitate care transfer and coordinate simultaneous care between different healthcare organizations.  Quality improvement process: The implementation of policies and procedures that provide feedback on structures and practices enabling the improvement of the clinical quality of care and ensuring safety.  Clinical information management: The coherence of the methods and systems to capture, update, retrieve and monitor patient data in a timely, relevant and confidential manner.  Physical environment: Refers to the physical spaces where health services are provided. Notably, space, comfort, cleanliness, and clarity of informational signage to facilitate patient orientation in healthcare facilities are considered important sources of satisfaction. |
| **Advocacy** | Advocacy – The degree to which clinicians defend the best interests of individual patients and patient groups in terms of health (including social determinants of health) and healthcare. | *"Not sure it is clear between advocacy and militancy. I see a difference. We can fight for a patient (defend the right to a service, for example) or take a public stance (advocate). I would clarify what your definition of advocacy means."* | Advocacy – The degree to which clinicians defend the best interests of individual patients and patient groups in terms of health (including social determinants of health) and healthcare. This advocacy does not necessarily require a militant or public stance, but is expressed through a professional and ethical commitment to promoting patient well-being. |
| **Family context** | Family context – Family-centred care: The degree to which the clinician takes into account the family (in all its forms) and understands its influence on a person's health, engaging it as a partner in ongoing healthcare. | *"The term 'family' could be somewhat restrictive; consider a term such as 'support system', significant others, etc."* | Family and significant others-centred care: The degree to which the clinician takes into account the family (in all its forms) and significant others, and understands their influence on a person's health, engaging them as partners in ongoing healthcare. |
| **Community context** | Community context, as a construct, is defined by the combination of the following two definitions:  Community patient participation: The involvement of clients and community members in decisions regarding the structure of the clinic and the services provided (e.g., advisory committees, community governance).  Consideration of the population context: The degree to which primary care clinicians assess and respond to the health needs of the population they serve. | *"It is important to go beyond participation/consultation and to prioritize the partnership approach, which is based on the complementarity of professional knowledge and lived-experience expertise (e.g., living with illness, etc.)."*  *"I would change the term client to citizen. The population context is directly related to knowledge of the area served by professionals as well as the socio-demographic reality of the area."* | Community context, as a construct, is defined by the combination of the following two definitions:  Community patient participation: Community involvement in decisions about the structure of the clinic and services provided, through a partnership approach that values the complementarity of professional and experiential knowledge.  Consideration of the population context: The degree to which primary care clinicians assess and respond to the health needs of the population they serve. |
| **Relational aspects** | Relational aspects, as a construct, is defined by the combination of the following two definitions:  Respect: The degree to which health professionals and support staff meet users' expectations regarding interpersonal treatment, show respect for patients' dignity, and ensure adequate confidentiality.  Trust: The level of trust that patients give to health professionals. | *"Difficult to understand the term 'interpersonal treatment'."* | Relational aspects, as a construct, is defined by the combination of the following two definitions:  Respect: The degree to which health professionals and support staff meet users' expectations regarding human relations, show respect for patients' dignity, and ensure adequate confidentiality.  Trust: The level of trust that patients give to health professionals. |
| **Financial aspects** | Financial aspects, as a construct, is defined by the combination of the following two definitions:  Efficiency/productivity: Achieving the desired outcomes with the most cost-effective use of resources.  Financial access: Assessment of the amount the patient pays for medical consultations and prescribed treatment. | *–* | Financial aspects, as a construct, is defined by the combination of the following two definitions:  Efficiency/productivity: Achieving the desired outcomes with the most cost-effective use of resources.  Financial access: Assessment of the amount the patient pays for medical consultations and prescribed treatment. |
| **Team** | Team (clinical and non-clinical), as a construct, is defined by the combination of the following two definitions:  Intersectoral team: The degree to which the primary care clinician collaborates with practitioners from sectors other than health to provide services with a health impact.  Multidisciplinary team: Practitioners from various health disciplines collaborate to provide continuous care. | *"Add recognition of the patient as an expert in living with illness and therefore as a member of the team (e.g. multidisciplinary)."* | Team (clinical and non-clinical), as a construct, is defined by the combination of the following two definitions:  Intersectoral team: The degree to which the primary care clinician collaborates with practitioners from sectors other than health to provide services with a health impact.  Multidisciplinary team: Practitioners from various health disciplines collaborate with the patient, considered as an active partner in their care, to provide continuous and tailored care. |
| **Patient engagement** | Patient engagement – The desire and ability to choose to actively participate in care in a way specifically adapted to each individual, in cooperation with a care provider or institution, with the aim of maximizing outcomes or improving the care experience. | *"Replace 'cooperation' with 'partnership'."*  *"Add a dimension of recognition of the patient's knowledge and practice in their own care."* | Patient engagement is defined as a process of individual or organizational transformation that enables the patient and their caregivers to enter into relationship with different health actors. (DCPP and CIO-UDEM, 2016)  Patient engagement corresponds to the desire and ability of the person to actively choose their level of participation in care in their own way, in cooperation with a healthcare provider or institution, with the aim of maximizing their outcomes or improving their care experiences. (Higgins T, Larson E, and Schnall R, 2017) |
| **Overall satisfaction** | Overall satisfaction – Feeling of security and having adequate follow-up, as well as satisfaction with emotional support, health information, decision-making control, and the competencies of health professionals. | *–* | Overall satisfaction – Feeling of security and having adequate follow-up, as well as satisfaction with emotional support, health information, decision-making control, and the competencies of health professionals. |
| **General comments** |  | *"The use of the term 'construct' is barely comprehensible for the general public."*  *"Modification of the following sentence: Construct X takes shape through the association of X following definitions."*  *"Modification of certain questions: To what extent do you agree with including construct X in the measurement of patient experience in primary care?"*  *"Translate Comprehensiveness as Intégralité rather than Exhaustivité."* | Addition of a definition of the term construct at the beginning of the questionnaire:  [Construct, e.g. Accessibility], as a construct, is defined by the combination of the following [N] definitions:  Question wording revised to: "To what extent do you agree with including X (e.g. accessibility) in the measurement of patient experience in primary care?"  Comprehensiveness of services definition retained using the term Comprehensiveness. |
